# Supplementary material for: Parallel transmit pulse design for saturation homogeneity (PUSH) for magnetization transfer imaging at 7T
Source: Magn Reson Med. 2022 Mar 10;88(1):180–94. doi: 10.1002/mrm.29199 (PMC9315051; doi:10.1002/mrm.29199)
Supplement: Supplementary file 1 — Figure S1. Mean squared B1+ (B1+2=β2) of each sub‐pulse for the PUSH‐1, ‐2 and ‐3 pulses optimized in the Simulations section 3.2 (middle axial slice, β=1μT). The first three columns show the sub‐pulse B1+2, whereas the last column shows the total B1+2 which is the sum of the contributions from all sub‐pulses. For PUSH‐2 and ‐3 the sub‐pulses are highly complementary, yielding very uniform total B1+2. Figure S2. MTR simulations assuming an ideal homogeneous saturation pulse whilst alternately changing flip angle α and B1rms βex properties of the excitation pulse from spatially inhomogeneous to homogeneous. Simulations using target flip angle of (A) 5∘ and (B) 15∘, showing that for 5∘ any inhomogeneity in either α or βex induces small changes in MTR, whereas for 15∘ the induced changes are much larger, with α inhomogeneity being the largest confound. In these simulations the pattern from CP mode was used for the inhomogeneous profiles. Figure S3. NRMSE of B1rms for the axial slice positioned as indicated by the red line in the sagittal plane in (B), comparing CP mode with the optimized PUSH solutions using 1, 2 and 3 sub‐pulses (curves for 2 and 3 sub‐pulses are superimposed due to nearly identical performance). The gray area represents β where CP mode reached the local SAR limits and its voltage is capped. Slice 12 corresponds to the solution in Figure 2A. To navigate through different slices this document needs to be open on a JavaScript‐supporting PDF viewer, such as Adobe Acrobat Reader. Figure S4. Corresponding 2D B1rms maps for the MTR maps in Figure 6. Different rows correspond to different pulses (top row: CP mode; middle row: PUSH‐1; bottom row: PUSH‐2) and columns correspond to different β (increasing from left to right). Below each is the mean ± standard deviation of B1rms over the white matter mask also used in Figure 6. Figure S5. Corresponding B1rms violin plot distributions for the MTR data in Figure 7. B1rms distributions for saturation pre‐pulses des [file MRM-88-180-s001.pdf]

Parallel transmit PUlse design for Saturation Homogeneity (PUSH) for Magnetization Transfer imaging at 7T: Supporting Information

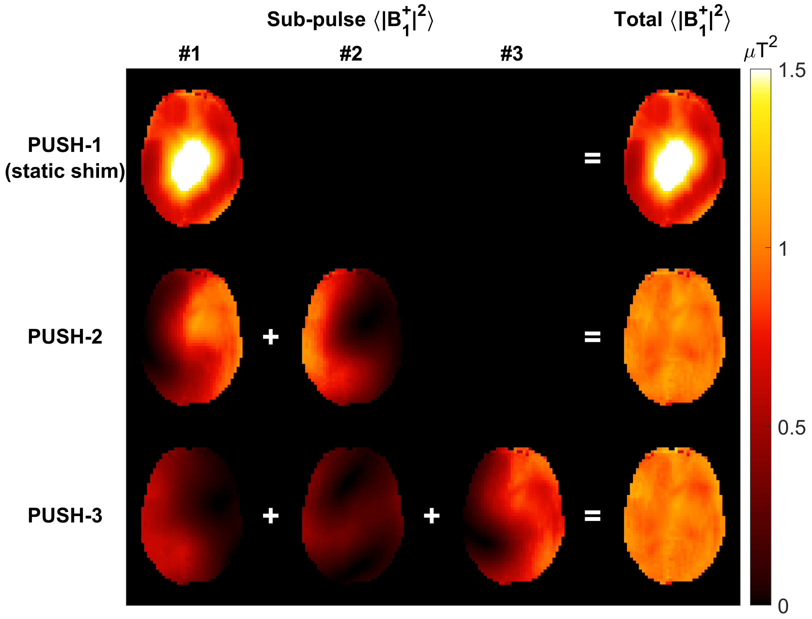

Supporting Information Figure S1: Mean squared B_1^+ ($\langle|B_1^+|^2\rangle = \beta^2$) of each sub-pulse for the PUSH-1, -2 and -3 pulses optimized in the Simulations section 3.2 (middle axial slice, $\beta = 1\mu T$). The first three columns show the sub-pulse $\langle|B_1^+|^2\rangle$, whereas the last column shows the total $\langle|B_1^+|^2\rangle$ which is the sum of the contributions from all sub-pulses. For PUSH-2 and -3 the sub-pulses are highly complementary, yielding very uniform total $\langle|B_1^+|^2\rangle$.

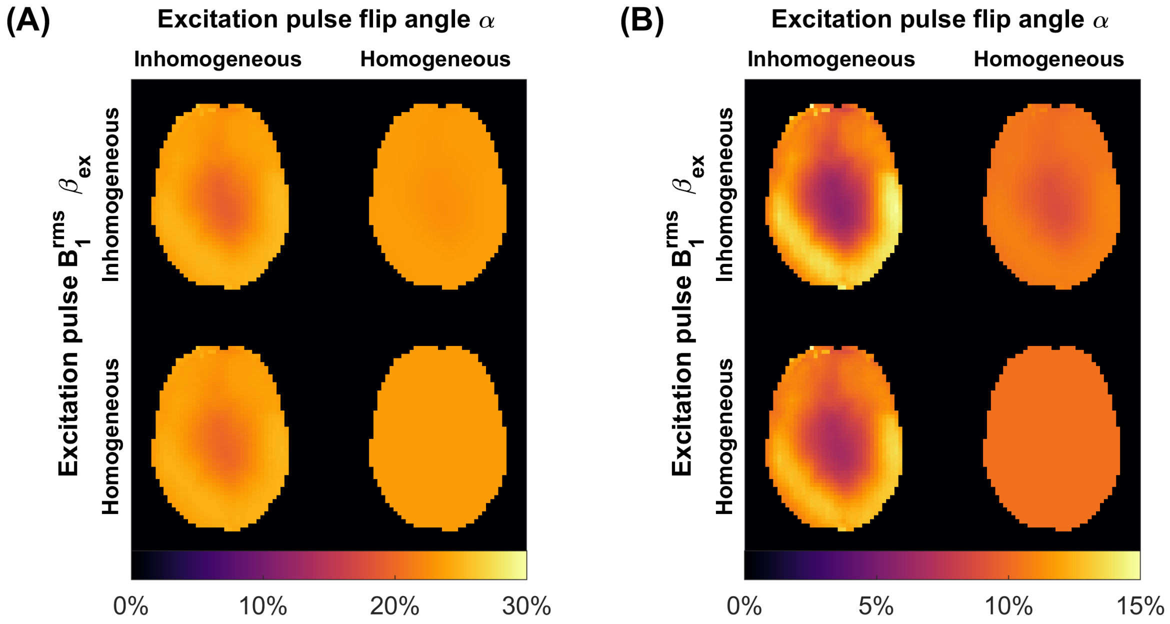

Supporting Information Figure S2: MTR simulations assuming an ideal homogeneous saturation pulse whilst alternately changing flip angle α and B_1^{rms} β_{ex} properties of the excitation pulse from spatially inhomogeneous to homogeneous. Simulations using target flip angle of (A) 5° and (B) 15°, showing that for 5° any inhomogeneity in either α or β_{ex} induces small changes in MTR, whereas for 15° the induced changes are much larger, with α inhomogeneity being the largest confound. In these simulations the pattern from CP mode was used for the inhomogeneous profiles.

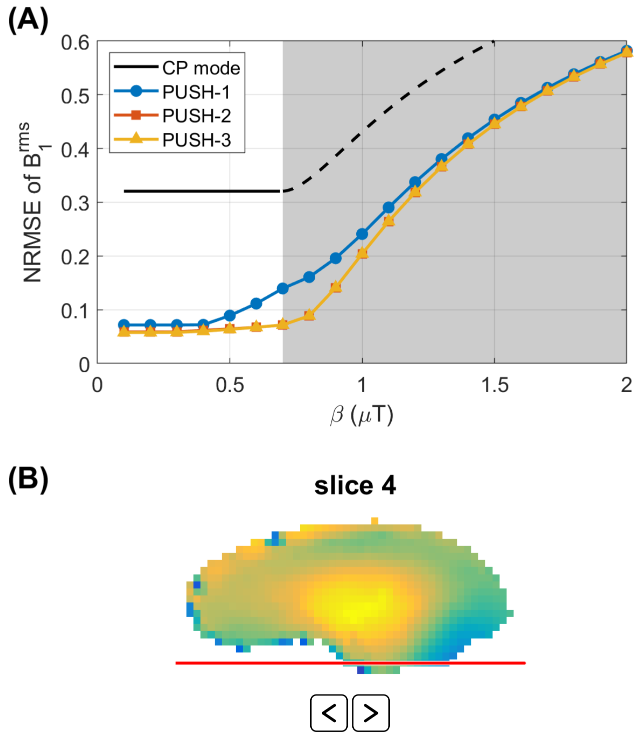

Supporting Information Figure S3: (A) NRMSE of B_1^{rms} for the axial slice positioned as indicated by the red line in the sagittal plane in (B), comparing CP mode with the optimized PUSH solutions using 1, 2 and 3 sub-pulses (curves for 2 and 3 sub-pulses are superimposed due to nearly identical performance). The gray area represents β where CP mode reached the local SAR limits and its voltage is capped. Slice 12 corresponds to the solution in Figure 2A. To navigate through different slices this document needs to be open on a JavaScript-supporting PDF viewer, such as Adobe Acrobat Reader.

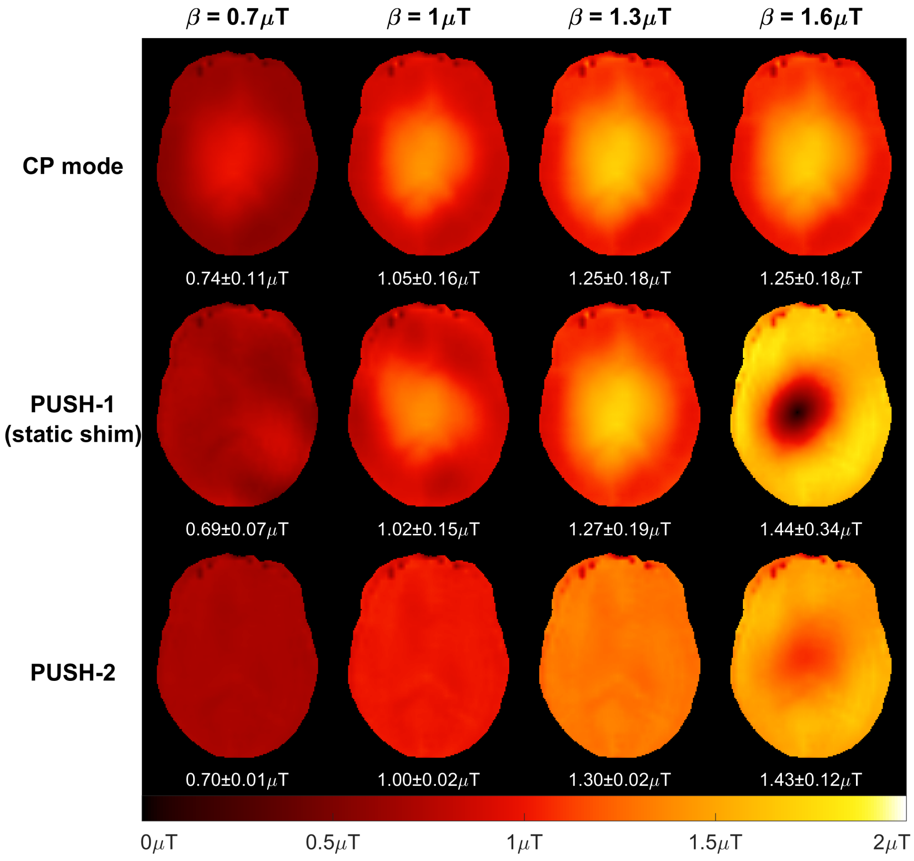

Supporting Information Figure S4: Corresponding 2D B_1^{rms} maps for the MTR maps in Figure 6. Different rows correspond to different pulses (top row: CP mode; middle row: PUSH-1; bottom row: PUSH-2) and columns correspond to different β (increasing from left to right). Below each is the mean ± standard deviation of B_1^{rms} over the white matter mask also used in Figure 6.

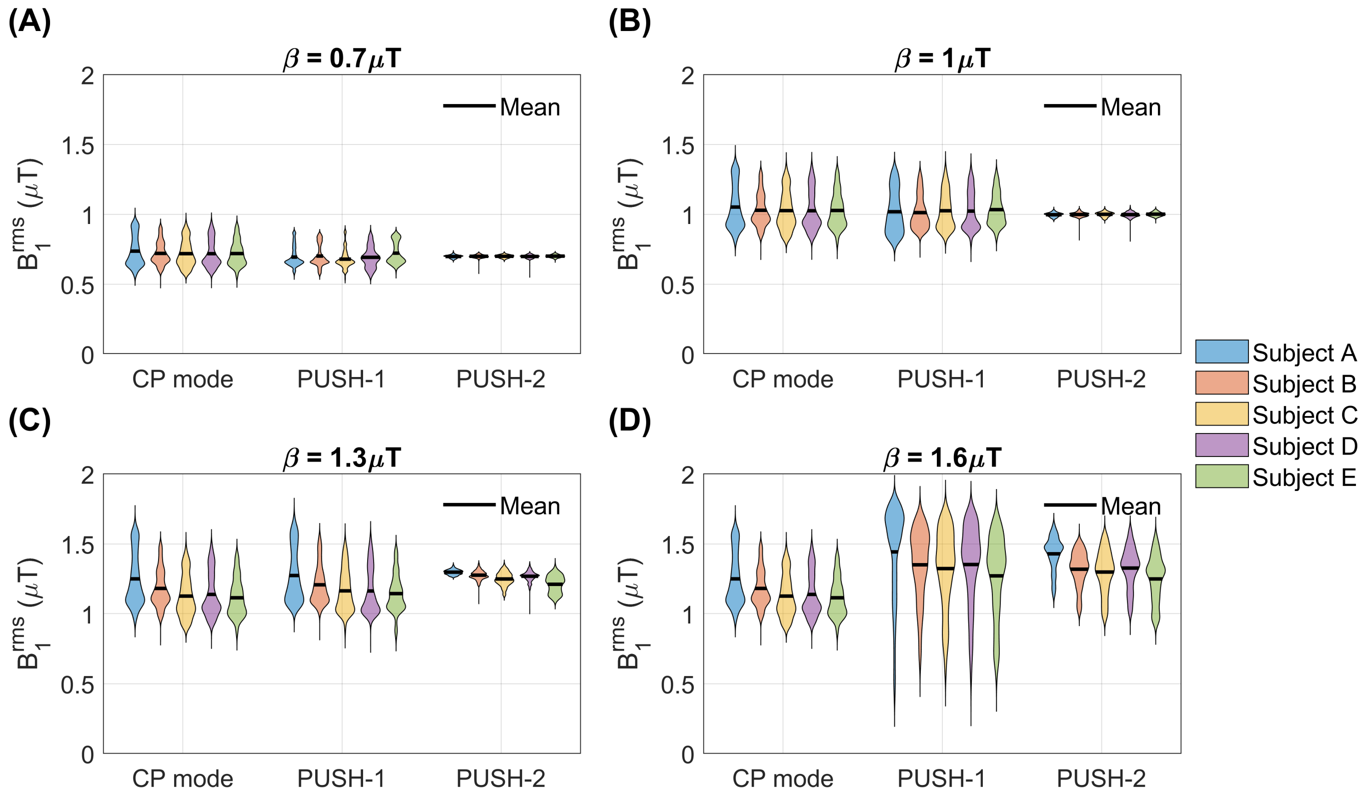

Supporting Information Figure S5: Corresponding B_1^{rms} violin plot distributions for the MTR data in Figure 7. B_1^{rms} distributions for saturation pre-pulses designed using β of (A) $0.7\mu T$, (B) $1\mu T$, (C) $1.3\mu T$ and (D) $1.6\mu T$. The black line represents the mean B_1^{rms}. Subjects are sorted in increasing order of reference voltage, which is inversely proportional to the maximum β achievable with CP mode.

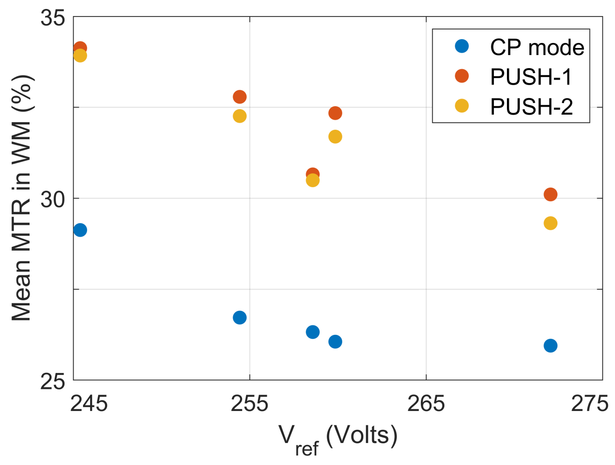

Supporting Information Figure S6: Mean MTR in WM ($\beta = 1.6\mu T$) versus the reference voltage V_{ref} associated to each subject. At the highest β all pulses are at the SAR limits and the mean MTR is indicative of the maximum MTR achieved.

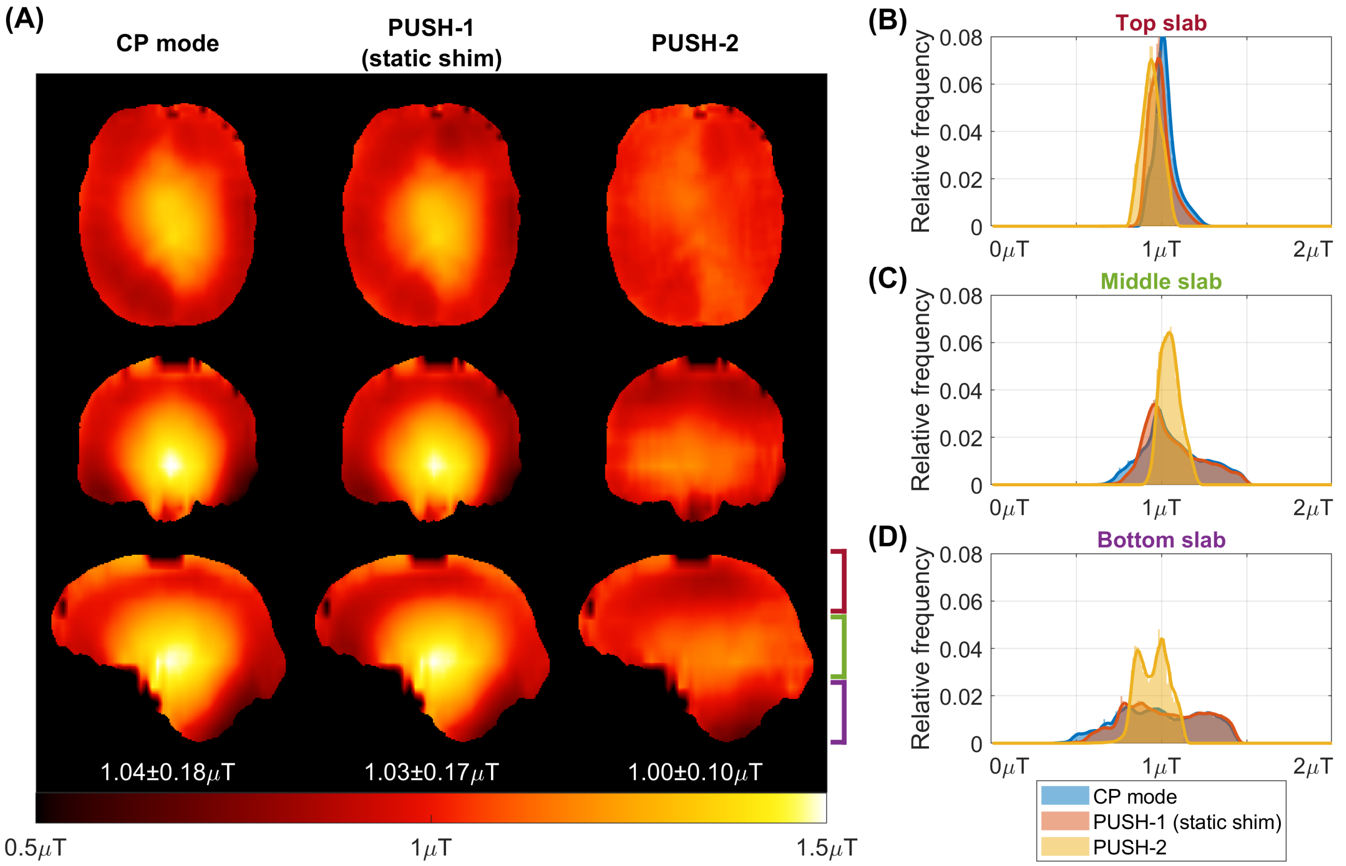

Supporting Information Figure S7: Corresponding B_1^{rms} for the MTR data in Figure 8. (A) Transverse, coronal and sagittal planes of the 3D B_1^{rms} maps. The left column contains the B_1^{rms} maps using CP mode, middle column using PUSH-1 and right column using PUSH-2. Below each sagittal plane is the mean ± standard deviation of B_1^{rms} over the white matter mask also used in Figure 8. (B-D) Histograms of the B_1^{rms} distribution in white matter over three slabs: (B) top slab, (C) middle slab, and (D) bottom slab, as illustrated in (A) near the bottom right sagittal plane. Moving average plotted jointly with histograms to delineate distribution trend.

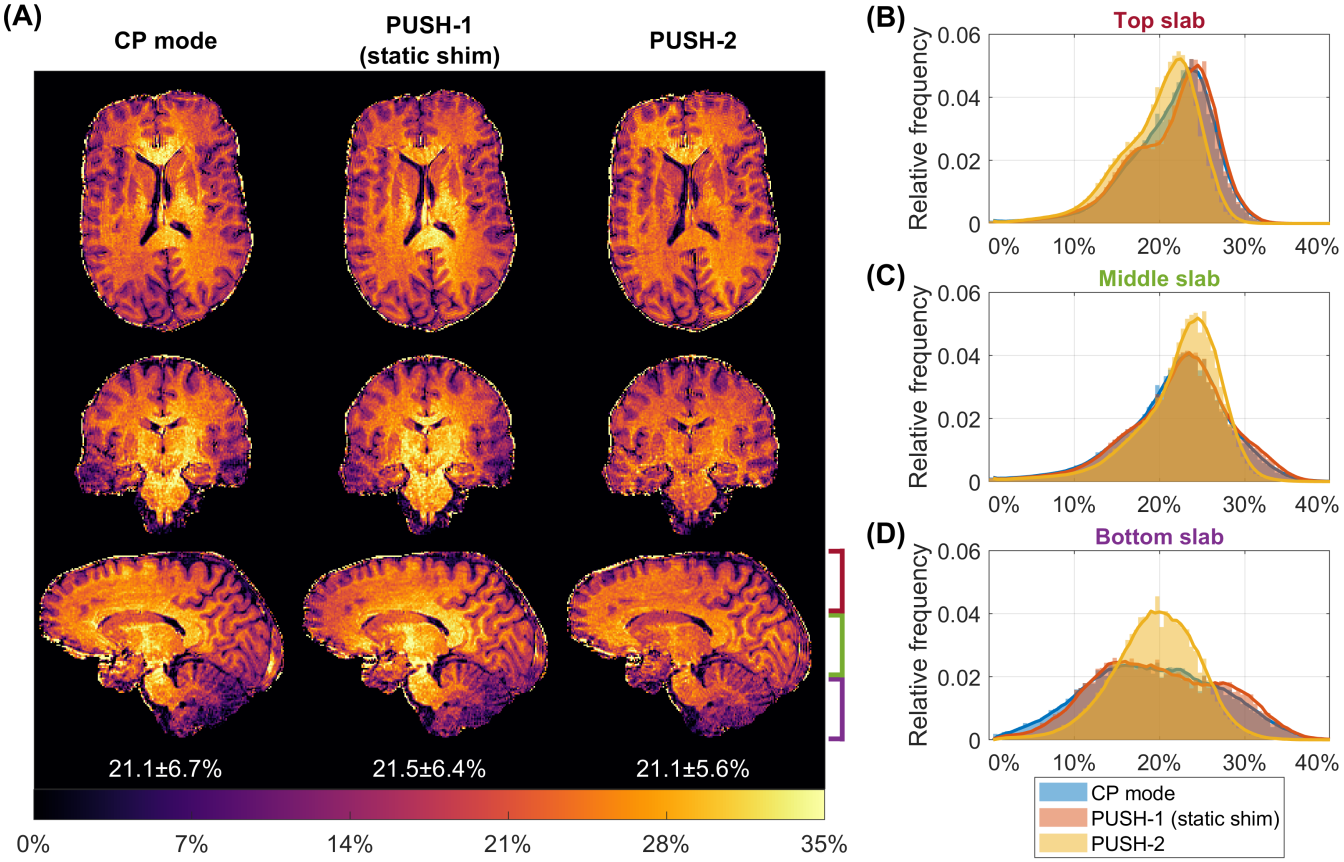

Supporting Information Figure S8: (A) Transverse, coronal and sagittal planes of the 3D MTR maps for subject E ($\beta = 1\mu T$). The left column contains the MTR maps acquired using CP mode, middle column using PUSH-1 and right column using PUSH-2. Below each sagittal plane is the mean ± standard deviation of MTR over the white matter mask. (B-D) Histograms of the MTR distribution in white matter over three slabs: (B) top slab, (C) middle slab, and (D) bottom slab, as illustrated in (A) near the bottom right sagittal plane. Moving average plotted jointly with histograms to delineate distribution trend.

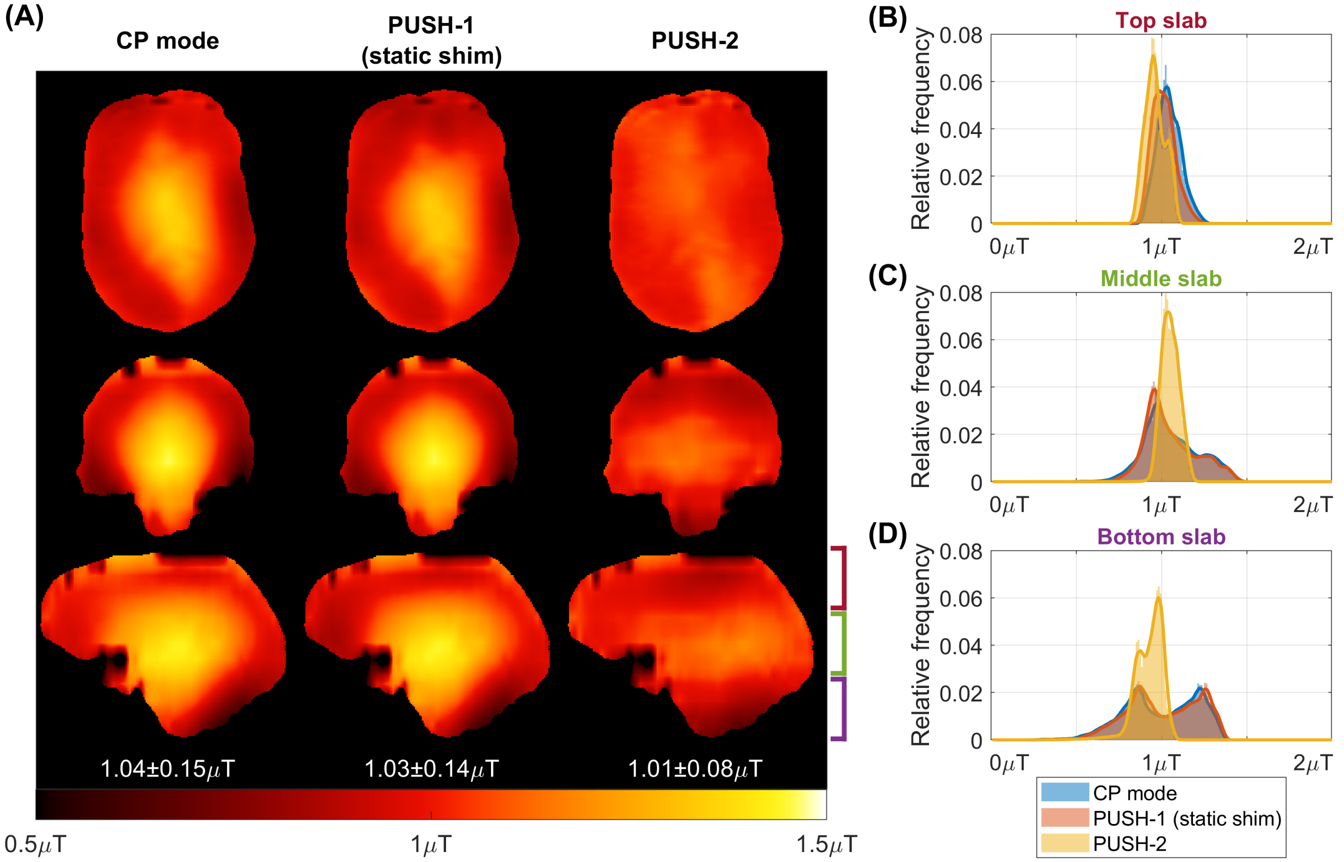

Supporting Information Figure S9: Corresponding B_1^{rms} for the MTR data in Supporting Information Figure S8. (A) Transverse, coronal and sagittal planes of the 3D B_1^{rms} maps. The left column contains the B_1^{rms} maps using CP mode, middle column using PUSH-1 and right column using PUSH-2. Below each sagittal plane is the mean $\pm$ standard deviation of B_1^{rms} over the white matter mask also used in Supporting Information Figure S8. (B-D) Histograms of the B_1^{rms} distribution in white matter over three slabs: (B) top slab, (C) middle slab, and (D) bottom slab, as illustrated in (A) near the bottom right sagittal plane. Moving average plotted jointly with histograms to delineate distribution trend.
